# Interactions between blending and identity concealment: Effects on non-binary people’s distress and experiences of victimization

**DOI:** 10.1371/journal.pone.0248970

**Published:** 2021-03-19

**Authors:** Sana Flynn, Nathan Grant Smith

**Affiliations:** Department of Psychological, Health, and Learning Sciences, College of Education, University of Houston, Houston, Texas, United States of America; Hackensack Meridian Health, UNITED STATES

## Abstract

Identity concealment (whether or not a person is open with others about their transgender status) and passing/blending (how much a transgender person can, or chooses to, blend into the binary social environment) have been shown to impact transgender people’s experiences in various ways, but few studies examine these constructs in the lives of non-binary individuals (those whose gender identity does not fall exclusively into the categories of man or woman). This study analyzed the non-binary subset of the nationwide sample from the 2015 United States Transgender Survey (9,769 participants) to examine the effects of blending/passing and identity concealment on distress and victimization. When ethnicity and income were controlled for, low concealers reported higher distress and more victimization experiences than high concealers, and blenders reported more distress and fewer victimization experiences than non-blenders. Not concealing may put non-binary people at higher risk for victimization, but blending into the binary-gendered environment may increase distress through identity erasure. Implications are discussed and future research directions are suggested.

## Introduction

According to the United States Transgender Survey (2015), around one-third of transgender people identify as a gender identity outside of the traditional man/woman binary [1]. There are a variety of labels that they use, including but not limited to genderqueer, agender, bigender, and genderfluid. Some conceptualize their gender as belonging between man and woman, and others as outside the man/woman continuum entirely [2]. For the purposes of the present study, “non-binary” will be used to refer to individuals who identify as a gender other than man or woman. Although people who identify as non-binary do not always use the term transgender to describe themselves, non-binary gender is considered a subset under the transgender umbrella in the current research.

According to the United States Transgender Survey [1], the largest survey to date examining the experiences of transgender people, 35% of respondents identified as non-binary. Although they make up around one-third of the overall transgender community [1], non-binary people are rarely studied as a discrete population, despite the possibility that their experiences may be different from transgender people who identify within the binary [3]. The present study seeks to address the lack of research on this population by examining an entirely non-binary sample.

A disproportionate number of mental health disparities have been observed in the transgender population, including inflated rates of Post-Traumatic Stress Disorder [4], eating disorders [5], general psychological distress, and suicidal thoughts and behaviors [1]. According to the United States Transgender Survey, 39% of respondents reported that they were currently experiencing psychological distress, significantly higher than the 5% rate in the United States population. Of those who were experiencing psychological distress, 29% reported that it interfered with their daily activities “a lot.” This percent is more than double the rate of the United States general population (12%). Moreover, 80% of transgender respondents reported having had suicidal thoughts in their lifetime, while 40% reported having attempted suicide at least once [1]. This is almost 10 times the rate of suicide attempts in the general population (4.6%) [1].

Recent studies have focused on examining variables that may contribute to suicidality, such as victimization [6] and minority stress [7]. Meyer [8] theorized that social stress specific to LGBQ people living in a society that enforces heterosexuality as the norm is responsible for mental health disparities in this community. However, transgender people can have different experiences relating to gender minority status beyond the discrimination experienced by sexual minorities, including barriers to accessing medical care and public restrooms [9, 10]. Testa and colleagues [9] expanded Meyer’s theory to develop the Gender Minority Stress Theory, which differs from Meyer’s model in several key ways to accommodate transgender people’s unique struggles (e.g., non-affirmation of gender identity, reflecting experiences where the social world fails to affirm the individual’s sense of gender identity by using inappropriate pronouns, honorifics, or given name).

### Identity concealment

Societal stigma and microaggressions have been demonstrated to have a negative impact on mental health for people with minority identities, but not all such identities are immediately visible. A concealable stigmatized identity is an identity that has the potential to be hidden from others [11]. A study by Quinn, Weisz, and Lawner [12] found that active concealment of a stigmatized identity in a mixed sample of participants with chronic illnesses, mental illnesses, or sexual minority status was negatively correlated with quality of life. They suggested that it is the frequent use of concealment strategies to hide the stigmatized identity that has a negative effect on quality of life.

In a study of participants with a chronic illness, Cook, Salter, and Stadler [13] found a relationship between concealment of chronic illness and employment status, with those concealing their chronic illness more likely to be employed. They suggested this may be due to avoidance of discrimination through concealment. At the same time, concealment was negatively associated with accessing social support; however, social support was positively correlated with positive health outcomes [13]. In a qualitative study on LGBTQ youth, Bry, Mustanski, Garofalo, and Burns [14] found a similarly complex picture of identity concealment. Some participants reported increased stigma, discrimination, and strained relationships after coming out, including more frequent negative comments about their sexual orientation or being subjected to sexual orientation change efforts (i.e., therapy aimed at converting a client’s sexual orientation to heterosexual). Conversely, others found that coming out actually decreased the microaggressions they received from their social networks and provided access to better social support, including financial and emotional support [14]. Martinez and colleagues [15] surveyed transgender employees and found that relational authenticity at work, or the degree to which participants were revealing and expressing their authentic gender identity, was positively associated with job satisfaction. However, participants also expressed that coming out could result in increased discrimination, as well as confusion and hostility from coworkers [15]. In other words, within populations with a concealable stigmatized identity, concealment can result in positive consequences by protecting the person from being socially stigmatized; on the other hand, it can have negative consequences through blocking authenticity and preventing intimacy in relationships. This study focuses on how these aspects of identity concealment may or may not apply to non-binary people.

### Blending

According to transgender community members, the ability to go unrecognized as transgender by the general public is known as *passing* or *blending*, with *passing* being a more outdated term [16]. The term *blending* will be used in the current study as it is emerging as more acceptable by the community. Blending is based on appearance, vocal tone, movement, and other gendered features of behavior. When a transgender person blends, they are able to exist in public spaces without strangers realizing that they are transgender. Gender in the United States has strictly-policed boundaries, and people who can be identified as gender non-conforming are often subject to ostracization or victimization [17]. Therefore, blending is considered by many to be the safer option [17]. In one study that examined the experiences of homeless transgender people who sought services at shelters, not blending was correlated with negative experiences such as being kicked out, being physically and sexually assaulted by staff and other homeless people, and feeling unsafe [18]. Not blending can result in worse victimization when the transgender person belongs to another marginalized group, for example a minority race or ethnic group or a lower socio-economic status [19]. Thus, for multiply marginalized persons, blending is even more of a safety issue [19].

While blending is thought to reduce victimization [17–19], blending may impact distress in ways that have yet to be examined. In the few studies on the construct of blending in transgender people’s lives, participants have identified blending as empowering and affirming, expressing the transgender person’s rejection of their sex assigned at birth and embracing of their true gender [16]. On the other hand, blending can be experienced as inauthentic when the social world is not recognizing the complexity of a person’s transgender experience [16]. Indeed, once they become socially recognized as their gender, many report feeling better and less concerned with their gender identity than before their transition [16], likely because they are able to express their authentic selves [15]. Additionally, they experience less discrimination once their transgender status is no longer obvious to the people around them [20]. For non-binary people, blending as either a man or a woman can feel like erasure of non-binary identity [21], and may be a source of distress for some. Not blending, while it can endanger safety, may feel most congruent [22]. For some, blending is not their focus, and they report not being concerned with how others identify them [23]. The diversity of experiences in the non-binary community related to blending, along with a lack of research, make it difficult to predict whether blending is a major source of distress.

Emerging research suggests that non-binary people may experience unique microaggressions [24] and, compared to binary transgender people, report higher rates of victimization [25], and worse health outcomes [3]. Gaining a greater understanding of the factors that may contribute to health disparities for non-binary people could aid in developing strategies to reduce such health disparities. Learning more about how blending and identity concealment may impact distress as well as victimization in non-binary people will add significantly to the literature on this under-studied population.

## Method

### Data

The data for this study come from the United States Transgender Survey, the largest survey conducted to date on transgender people’s experiences in the United States [1]. The Survey was developed by a team of researchers and advocates with input from members of the transgender community. Participants were recruited through LGBTQ- and transgender-specific organizations, health centers, and support groups, in addition to online communities. Outreach to these supporting organizations was essential in order to reach people who are less likely to have access to the internet, including seniors, people of color, people of low socio-economic status, and those residing in rural areas. Approximately 400 organizations helped to recruit participants by advertising and endorsing the survey, with some organizations providing access to the survey on site or hosting events to encourage participants to take the survey. The survey was also promoted through a website, online platforms such as Facebook and Twitter, and articles, blogs, and op-eds. Data were collected through the online survey during the summer of 2015. Written informed consent was obtained at the beginning of the survey. Participants who entered a birthday indicating that they were under 18 were removed from the dataset during the data cleaning process. The survey contained questions relating to diverse topics such as social support, encounters with legal entities, access to public services, mental and physical health, and victimization. After completing the survey, participants were given the opportunity to enter into a cash prize drawing for a $500 prize or one of two $250 prizes. The original study was approved through the University of California, Los Angeles Institutional Review Board. The original sample includes 27,715 participants aged 18 or older who identified under the transgender umbrella and came from all US states and territories. Respondents were not randomly sampled, so the data cannot be generalized to all transgender people in the United States.

### Participants

Participants in the United States Transgender Survey [1] were asked “If you had to choose only one of the following terms, which best describes your current gender identity? (Please choose only one answer).” Response options included Cross-dresser, Woman, Man, Trans woman (MTF), Trans man (FTM), and Non-Binary/Genderqueer. For the current study, the subset of 9,769 participants who identified with the gender label “Non-Binary/Genderqueer” were used for analyses. See Table 1 for demographic information on this sample of 9,769. Participants primarily identified as White and of low socio-economic status, and most were between the ages of 18–24.

**Table 1:** Demographic characteristics of the sample.

Demographic	Value	Frequency	Percent
**Age**	18 to 24	5947	60.9
	25 to 44	3305	33.8
	45 to 64	458	4.7
	65 +	59	.6
	*M* = 25.64, *SD* = 9.096		
**Race/Ethnicity**	Alaska Native/ American Indian	93	1.0
	Asian/Native Hawaiian/Pacific Islander	332	3.4
	Biracial/Multiracial/Not Listed	700	7.2
	Black/African American	262	2.7
	Latinx/Hispanic	529	5.4
	White	7798	79.8
	Middle Eastern/North African	55	.6
**Census Region**	Northeast	2151	22.0
	Midwest	1992	20.4
	South	2579	26.4
	West	3027	31.0
	No Census Region	20	.2
**Household Income**	No Income	409	4.2
	$1 to $4,999	654	6.7
	$5,000 to $7,499	362	3.7
	$7,500 to $9,999	293	3.0
	$10,000 to $12,499	383	3.9
	$12,500 to $14,999	292	3.0
	$15,000 to $17,499	260	2.7
	$20,000 to $24,999	562	5.8
	$25,000 to $29,999	450	4.6
	$30,000 to $34,999	487	5.0
	$35,000 to $39,999	416	4.3
	$40,000 to $49,999	617	6.3
	$50,000 to $59,999	614	6.3
	$60,000 to $74,999	704	7.2
	$75,000 to $99,999	727	7.4
	$100,000 to $149,999	767	7.9
	$150,000 +	529	5.4
	Excluded*	263	2.7
	Missing	746	7.6

*Note*. Data were excluded from the Household Income recode in the original dataset if responses to questions regarding family size and income were illogical (e.g., if individual income was reported as greater than household income).

### Measures

#### Identity concealment

For the Identity Concealment independent variable, responses to the question “When people in your life assume you are something other than non-binary/genderqueer (such as a man or a woman), how do you respond?” were analyzed. Participants who answered “I usually let them assume I am a man or a woman” were considered high concealers. Participants who answered “I sometimes tell them I identify as non-binary/genderqueer (or whatever words I use)” or “I always tell them I identify as non-binary/genderqueer (or whatever words I use)” were considered low concealers.

#### Blending

For the Blending independent variable, responses to the statement “People can tell I’m trans even if I don’t tell them” (note that “trans” is a shortened version of “transgender” and is commonly used) were analyzed. Participants who responded with either “Rarely” or “Never” were considered blenders in the analysis. Participants who responded “Sometimes” “Most of the time” or “Always” were considered non-blenders.

#### Victimization

For the Victimization variable, all “Yes” responses to six questions about recent victimization experiences were added together. The questions were “In the past year, have you been denied equal treatment or service, such as at a place of business, government agency, or public place for any reason?,” “In the past year, did anyone verbally harass you for any reason?,” “In the past year, did anyone physically attack you (such as grab you, throw something at you, punch you, use a weapon) for any reason?,” “In the past year, did strangers verbally harass you in public because of your trans status, gender identity, or gender expression?,” “In the past year, did strangers physically attack you in public because of your trans status, gender identity, or gender expression?,” and “Now just thinking about the past year, have you experienced unwanted sexual contact (such as oral, genital, or anal contact or penetration, forced fondling, rape)?”

#### Psychological distress

For the distress variable, the original survey included the Kessler-6 Scale for psychological distress, a short form scale designed for measuring prevalence of mental health issues in large-scale health surveys [26]. The Kessler-6 has undergone extensive validity and reliability testing (α = .89 reported in literature; α = .63 in sample) and is used in government health surveys. Respondents were asked how frequently in the past 30 days they felt so sad that nothing could cheer them up, nervous, restless or fidgety, hopeless, that everything was an effort, or worthless. They selected (0) *none of the time*, (1) *a little of the time*, (2) *some of the time*, (3) *most of the time*, or (4) *all of the time* for each question. A score between 0 and 24 was created and this score was used to reflect level of psychological distress for this study.

#### Demographic covariates

Because both race/ethnicity and income were related to victimization in the original United States Transgender Survey report [1], these covariates were included in the analyses. Race/ethnicity was analyzed based on a recode in the original dataset that divided participants into seven categories: White, Alaska Native/American Indian, Asian/Pacific Islander, Biracial/Multiracial/Not listed, Black/African American, Latino/a/Hispanic, and Middle Eastern/North African. Race/ethnicity was dummy coded with White as the referent group because the majority of participants were White, and White participants were theorized to experience less victimization than other groups. In the current study, income was analyzed as a continuous variable using the total household income variable that captured 18 income ranges from no income to $150,000 or more.

### Statistical analysis

The identity concealment variable had two levels: low concealers and high concealers. The blending variable had two levels: blenders and non-blenders. The two independent variables created four subtypes of participants. These groups were compared in a two-way MANCOVA analysis to test for main and interaction effects on victimization and distress. Wilks’ lambda was examined with *p* < .05 considered significant. Univariate tests were conducted to examine between-groups main effects. The sample demographics were derived using descriptive statistics. Race/ethnicity and income were analyzed as covariates. All analyses were conducted using the Statistical Packages for the Social Sciences (SPSS) version 25.

## Results

Race/ethnicity and income were treated as covariates in the analysis. The MANCOVA revealed significant differences on the combined dependent variable between White and each of the following racial/ethnic groups: Alaska Native/American Indian (*F*_2, 9422_ = 7.894, Wilks’ *λ* = .998, *p* = .000, partial *η*^2^ = .002), Biracial/Multiracial/Not listed (*F*_2, 9422_ = 16.682, Wilks’ *λ* = .996, *p* = .000, partial *η*^2^ = .004), and Middle Eastern/North African (*F*_2, 9422_ = 5.341, Wilks’ *λ* = .999, *p* = .005, partial *η*^2^ = .001). Univariate *F* tests revealed that Alaska Native/American Indian (*F*_1, 9422_ = 14.191, *p* = .000, partial *η*^2^ = .002), Biracial/Multiracial/Not listed (*F*_1, 9422_ = 33.310, *p* = .000, partial *η*^2^ = .004), and Middle Eastern/North African (*F*_1, 9422_ = 9.454, *p* = .002, partial *η*^2^ = .001) groups all reported more victimization than White participants, but there were no significant differences on distress based on race/ethnicity. See Table 2 for means and standard deviations of the race/ethnicity categories. Similarly, the MANCOVA results showed a significant effect of household income on victimization (*F*_1, 9423_ = 11.694, *p* = .001, partial *η*^2^ = .001) but not on distress (*F*_1, 9423_ = .306, *p* = .580, partial *η*^2^ = .000). Participants who reported lower household income reported more victimization experiences, *r*(9767) = -.05, *p* = .000.

**Table 2:** Means for distress and victimization by racial/ethnic group.

Racial or Ethnic Group	*n*	Distress*	*SD*	Victimization**	*SD*
**Alaska Native/American Indian**	93	15.516	13.788	1.979	1.608
**Asian/Native Hawaiian/Pacific Islander**	332	12.937	11.119	1.464	1.465
**Biracial/Multiracial/Not Listed**	700	13.654	11.776	1.769	1.493
**Black/African American**	262	13.905	12.030	1.573	1.378
**Latinx/Hispanic**	529	14.214	11.834	1.459	1.389
**Middle Eastern/North African**	55	15.673	17.063	2.073	1.345
**White**	7798	13.334	12.264	1.427	1.343
**Total**	9769	13.445	12.212	1.467	1.369

*Note: Range: 0–24

**Note: Range: 0–6

After controlling for race/ethnicity and income, the MANCOVA revealed significant main effects for identity concealment (*F*_2, 9422_ = 72.819, Wilks’ *λ* = .985, *p* = .000, partial *η*^2^ = .015) and blending (*F*_2, 9422_ = 148.869, Wilks’ *λ* = .969, *p* = .000, partial *η*^2^ = .031). The interaction effect was non-significant (*F*_2, 9422_ = .985, Wilks’ *λ* = 1.000, *p* = .373, partial *η*^2^ = .000). See Table 3 for a summary of the MANCOVA results.

**Table 3:** MANCOVA results.

	Wilks’ *λ*	*F*	*p*	partial *η*^2^
**Identity Concealment**	.985	72.819	.000	.015
**Blending**	.969	148.869	.000	.031
**Interaction**	1.000	.985	.373	.000
**Alaska Native/American Indian**	.998	7.894	.000	.002
**Asian/Native Hawaiian/Pacific Islander**	1.000	.580	.560	.000
**Biracial/Multiracial/Not Listed**	.996	16.682	.000	.004
**Black/African American**	.999	2.360	.094	.001
**Latinx/Hispanic**	1.000	1.581	.206	.000
**Middle Eastern/North African**	.999	5.341	.005	.001
**Household Income**	.999	5.867	.003	.001

Univariate *F* tests revealed that low concealers reported significantly more distress (*M* = 13.621, *SE* = .167) than high concealers (*M* = 13.050, *SE* = .208), *F*_1, 9423_ = 4.584, *p* = .032, partial *η*^2^ = .000. Low concealers reported more victimization (*M* = 1.681, *SE* = .018) than high concealers (*M* = 1.330, *SE* = .023), *F*_1, 9423_ = 144.831, *p* = .000, partial *η*^*2*^ = .015. Blenders reported more distress (*M* = 13.742, *SE* = .160) than non-blenders (*M* = 12.929, *SE* = .214), *F*_1, 9423_ = 9.241, *p* = .002, partial *η*^*2*^ = .001. Finally, blenders (*M* = 1.263, *SE* = .018) reported fewer instances of victimization than non-blenders (*M* = 1.748, *SE* = .023), *F*_1, 9423_ = 274.992, *p* = .000, partial *η*^*2*^ = .028. See Table 4 for a summary of the univariate tests.

**Table 4:** Univariate tests results.

	*n*	Distress *M*	*F*	*p*	partial *η*^2^	Victimization *M*	*F*	*p*	partial *η*^2^
**Low Concealers**	5302	13.621	4.584	.032	.000	1.681	144.831	.000	.015
**High Concealers**	4132	13.050				1.330			
**Blenders**	5774	13.742	9.241	.002	.001	1.263	274.992	.000	.028
**Non-blenders**	3660	12.929				1.748			

## Discussion

This study contributes to the literature on non-binary transgender people by examining identity concealment and blending and how these constructs are related to distress and victimization. This study offers a first glance at the relationships among these variables in an understudied population, as well as confirming the significant negative correlation between blending and victimization seen in prior studies of binary transgender people [17–19].

Study results demonstrated that participants who did not blend reported more instances of victimization than participants who did blend, likely due to the fact that people who blend into a binary-gendered environment are less easy to identify as gender non-conforming by people who may target them. This finding is consistent with the literature on blending, supporting the claim that blending is connected with physical safety [18]. Low concealers also reported more victimization experiences. Being open about identifying as non-binary or genderqueer might make low concealers easier to target, similar to other LGBTQ populations that have been more-widely studied; in one study about sexual minority youth, coming out was associated with rejection and increases in microaggression experiences [14]. Non-binary people may experience more victimization when they are known to be gender non-conforming.

Regarding the outcome variable of distress, participants who blended reported higher distress than those who did not blend. These results may reflect a difference between the experiences of binary and non-binary transgender people. Binary transgender people report a sense of satisfaction when they are recognized as their gender identity by the general public [16]. However, non-binary people may not be able to blend as their gender in a binary-gendered environment where the choices are man or woman. Perhaps this higher level of distress reflects a lack of recognition of their non-binary gender by others. On the other hand, participants who did not blend may have reported less distress because not blending expresses their gender identity more accurately. For binary transgender people, congruence between their internal sense of their gender and their appearance (blending as their identified gender) can feel affirming [15]. For non-binary people, blending may feel erasing, while not blending (standing out from the binary gendered social environment) may be experienced as more congruent with non-binary gender. In other words, not blending may be one way to express a non-binary identity in social contexts, resulting in less distress.

Participants who were classified as low in identity concealment reported higher distress than high concealers. This finding seems to imply that being open about having a non-binary gender identity may be linked to greater distress. Non-binary people report that the communities to which they belong can struggle to understand the concept of non-binary gender identity [21]. Perhaps being out as non-binary, with the constant work of correcting pronouns or explaining gender, contributes to distress when navigating a world that is only accustomed to classifying people as men or women.

Overall, non-binary people who express themselves outside of the binary can find themselves in a double bind as they navigate authenticity and safety. On the one hand, being out can feel more congruent when social contacts recognize a person’s non-binary gender identity. However, attempting to educate social contacts about identities between or outside of the binary can be exhausting and distressing. Additionally, expressing non-binary identity by not blending can result in confusion and hostility from the general public, while conforming to binary gender expectations can feel erasing. Either way, non-binary people may face victimization or distress from any choice regarding gender identity expression.

Although most studies have conflated non-binary with binary transgender experience, the two distinct communities may have different relationships with the social world. Further investigation of non-binary people’s experiences may be useful to clinicians working with non-binary individuals, as gender identity concealment and blending impact social relationships. Non-binary individuals often need to balance authenticity with safety in both gender presentation and gender identity disclosure, a choice that must be made on a daily basis when existing in public spaces as well as in individual social relationships.

Moreover, the struggle of holding an identity that is unrecognized by the wider culture means that non-binary people frequently cope with microaggressions in many areas of their social spheres. Furthermore, when looking for belonging in the wider transgender community, non-binary people may have difficulty locating inclusive resources or relating to transgender people who subscribe to the binary view of gender. Clinicians must be aware of these unique circumstances, such as the lack of inclusivity in some transgender spaces, in order to understand social situations that non-binary clients may face and to determine if resources are appropriate.

### Limitations

The current dataset and findings have some important sample limitations. As an online survey, the United States Transgender Survey had the sample bias typical of online surveys, skewed towards White participants with internet access. Extensive recruitment efforts focused on reaching other demographics, particularly people of Color, mitigated the situation somewhat, but the sample still was largely White and thus is likely not representative of the larger population of non-binary persons. There were efforts to provide access to the survey to transgender people with no internet access through community centers in order to reach a wider diversity of people at different levels of income. In addition, the non-binary sample utilized in this study reported a wide variation in income that may not be indicative of non-binary individuals in general. One reason might be related to the age of the sample, which was primarily young adults. This age group may have reported family income due to living at home. Nevertheless, because the sample was not random, the results cannot be generalized to all non-binary transgender people [1].

Another limitation of this study is that the constructs of blending and identity concealment are defined only in terms of one question each. Future research should strive to investigate these constructs using more detailed questionnaires in order to further tease apart the possibly differential effects of blending and identity concealment on non-binary people’s experiences and well-being. For non-binary people, the construct of blending is potentially more complex than can be understood using a dichotomous variable. There may be significant differences between experiences of non-binary people who are blending as their gender assigned at birth and those who are blending as another gender. For example, blending as gender assigned at birth could potentially be experienced as more erasing for some. Future research should address the nuances of blending as a man or woman in relation to non-binary people’s gender assigned at birth.

Decisions to conceal a non-binary identity may be based heavily on context and situation—nuances that have not been captured by these data and were not explored in the current study. Likewise, different social environments may be more conducive to blending or not blending. While the two constructs both involve choice to some degree, such as decisions around appearance, blending is more focused on other peoples’ views, which is somewhat outside the control of the individual, whereas identity concealment is a more active process, over which the individual has more control. A non-binary individual may look the same in two different contexts, but be read differently by people in those two contexts. Additionally, non-binary people have a choice to actively conceal or reveal their gender identity in different ways. The same person may choose to present with a different gender expression depending on social context, shifting experience of gender (gender fluidity), mood, or geographic location for a variety of reasons. Choices about gender expression can range from personal preference to physical safety, and again, may be highly context-dependent. Future research should explore the contextual nature of choices to conceal as well as ability to blend.

Due to the cross-sectional nature of the study, the direction of any effects is not possible to discern. For example, victimization and distress may impact identity concealment and blending, or vice-versa. Additionally, the victimization experiences in the survey, such as sexual, physical, and verbal assault or harassment, occur for a variety of reasons and may not be caused by low concealment or non-blending. More research is needed to investigate the relationships between variables for this population.

This study provides a small window into common experiences of non-binary people. The data contain responses of over 9,000 non-binary individuals from diverse backgrounds and locations [1], the largest sample of non-binary people to date. This research is a starting point for more nuanced study of a population that is under-researched and often conflated with the larger transgender community. As the transgender community becomes more publicly visible and receives more attention from researchers, it is important not to leave non-binary people behind. Because they do not conform to the transgender narrative that has been used to make binary transgender people more acceptable to the general public, the non-binary community is often erased. This research may provide new directions for the study of this population’s relationship with the social world, assisting those who work with this population to understand health disparities and their unique experiences. Though these trends may not be generalizable to all non-binary people in the United States, this study is a beginning point for understanding a neglected segment of the gender non-conforming population.
